# Insertional Mutagenesis and Deep Profiling Reveals Gene Hierarchies and a *Myc/p53*-Dependent Bottleneck in Lymphomagenesis

**DOI:** 10.1371/journal.pgen.1004167

**Published:** 2014-02-27

**Authors:** Camille A. Huser, Kathryn L. Gilroy, Jeroen de Ridder, Anna Kilbey, Gillian Borland, Nancy Mackay, Alma Jenkins, Margaret Bell, Pawel Herzyk, Louise van der Weyden, David J. Adams, Alistair G. Rust, Ewan Cameron, James C. Neil

**Affiliations:** 1 Centre for Virus Research, Institute of Infection, Immunity and Inflammation, College of Medicine, Veterinary Medicine and Life Sciences, University of Glasgow, Glasgow, United Kingdom; 2 Delft Bioinformatics Lab, Faculty of EEMCS, TU Delft, Delft, The Netherlands; 3 Glasgow Polyomics, Institute of Molecular, Cell & Systems Biology, College of Medical, Veterinary & Life Sciences, University of Glasgow, Glasgow, United Kingdom; 4 Wellcome Trust Sanger Institute, Hinxton, Cambridge, United Kingdom; Cincinnati Children's Hospital Medical Center, United States of America

## Abstract

Retroviral insertional mutagenesis (RIM) is a powerful tool for cancer genomics that was combined in this study with deep sequencing (RIM/DS) to facilitate a comprehensive analysis of lymphoma progression. Transgenic mice expressing two potent collaborating oncogenes in the germ line (CD2-*MYC*, -*Runx2*) develop rapid onset tumours that can be accelerated and rendered polyclonal by neonatal Moloney murine leukaemia virus (MoMLV) infection. RIM/DS analysis of 28 polyclonal lymphomas identified 771 common insertion sites (CISs) defining a ‘progression network’ that encompassed a remarkably large fraction of known MoMLV target genes, with further strong indications of oncogenic selection above the background of MoMLV integration preference. Progression driven by RIM was characterised as a Darwinian process of clonal competition engaging proliferation control networks downstream of cytokine and T-cell receptor signalling. Enhancer mode activation accounted for the most efficiently selected CIS target genes, including *Ccr7* as the most prominent of a set of chemokine receptors driving paracrine growth stimulation and lymphoma dissemination. Another large target gene subset including candidate tumour suppressors was disrupted by intragenic insertions. A second RIM/DS screen comparing lymphomas of wild-type and parental transgenics showed that CD2-*MYC* tumours are virtually dependent on activation of *Runx* family genes in strong preference to other potent Myc collaborating genes (*Gfi1, Notch1*). *Ikzf1* was identified as a novel collaborating gene for *Runx2* and illustrated the interface between integration preference and oncogenic selection. Lymphoma target genes for MoMLV can be classified into (a) a small set of master regulators that confer self-renewal; overcoming p53 and other failsafe pathways and (b) a large group of progression genes that control autonomous proliferation in transformed cells. These findings provide insights into retroviral biology, human cancer genetics and the safety of vector-mediated gene therapy.

## Author Summary

Cancers are known to arise by a series of mutational and non-mutational (epigenetic) events but the advent of cancer genome sequencing highlights the growing challenge of separating important (driver) from irrelevant (passenger) mutations. Retroviruses that induce cancer by inserting into host DNA and thereby altering key genes are valuable tools because they act as ‘tags’ to identify the critical targets. In this study we combined retroviral tagging with next generation sequencing to achieve a comprehensive description of lymphoma development and progression in transgenic mouse model systems. Our study suggests that three events may be sufficient for lymphoma development and identifies a genetic bottleneck at a small gene set that regulates tumour cell self-renewal, including the *Myc* oncogene and the p53 tumour suppressor. In contrast, many genes can provide the final step where the lymphoma cell acquires the ability to divide independently of external stimuli. As many of the target genes are conserved and play roles in cancers of non-viral origin, this study may provide a paradigm for the gene interactions that underlie cancer biology. It also elucidates the risks entailed in the recent use of retrovirus-based vectors for human gene therapy.

## Introduction

The oncogenic potential of murine γ-retroviruses (MLVs) stems from proviral integration into host DNA, a mutagenic process which can result in activation or disruption of critical host cell genes [1]. Moreover, by sequential integrations in the nascent tumour cell, MLVs can drive multiple steps in the oncogenic process. These features have led to the use of MLVs as screening tools for genes relevant to cancer, particularly haematopoietic malignancies. The reach of this approach has grown considerably with the development of high throughput methods for cloning and sequencing analysis of host-virus junctions at insertion sites, facilitating screens of large tumour panels and identifying hundreds of genes of potential relevance to cancer. Importantly, genes identified by this method frequently map to orthologous sites of mutation in human cancer [2], [3]. Moreover, retroviral insertional mutagenesis (RIM) provides a complementary approach to whole genome sequencing and copy number analysis in cancer, as RIM has the potential to uncover genes that are rarely mutated but more commonly subject to indirect processes including epigenetic modification [4]. Furthermore, large scale analyses of co-occurrence of target genes can identify patterns indicating collaborative or redundant relationships between cancer genes [5], [6]. Despite the wealth of information provided by these studies, it is not yet known whether two events are sufficient for lymphoid transformation or whether higher order collaborations between more than two target genes are required. Target gene interactions can be explored functionally when combined with manipulation of the mouse genome and mice with an activated oncogene or mutant tumour suppressor gene in the germ-line often show accelerated tumour onset [7], [8]. RIM tagging in this context reveals preferential targeting of specific collaborating genes, which can be confirmed by analysis of compound transgenic mice [1].

Moloney murine leukaemia virus (MoMLV) is an oncogenic γ-retrovirus that has been widely used in RIM studies [3], [9], [10] and owes its potency to a duplicated enhancer element in the proviral long terminal repeats (LTRs) [11]. Notably, the LTRs and backbone of this virus formed the basis of retroviral vector systems used in early trials of human gene therapy, where leukaemia resulting from insertional activation of host genes has been a significant adverse outcome [12]. In mice, the target genes for MoMLV that have been identified to date show a predominance of oncogene activation events over tumour suppressor disruption, consistent with the observed low rate of loss of heterozygosity in MoMLV lymphomas [13]. However, these findings presented a long-standing puzzle in light of the effect of germ-line inactivation of the major tumour suppressor p53, which confers rapid onset T-cell lymphomas with a similar broad phenotypic spectrum to MoMLV but shows relatively weak cooperation with MoMLV [14]. We hypothesised previously that the MoMLV oncogenic programme must neutralise the tumour suppressor activity of p53, circumventing the need for direct mutations in the pathway [14], [15]. In support of this proposal we showed that the potent combination of two MoMLV target genes, *Myc* and *Runx2*, could overcome the need for genetic inactivation of the p53 pathway, despite the fact that both oncogenes evoke p53 growth suppression and collaborate strongly with p53 deficiency [16]. Nevertheless, this combination still appears to be insufficient for full transformation, as double transgenic tumours emerge as clonal outgrowths from a polyclonal premalignant phase [17]. We showed previously that tumour onset could be accelerated by retroviral infection and a RIM screen identified a number of candidate third hit genes, including *Pim1*, a gene that accelerates tumour onset when combined with *MYC/Runx2* in the germ-line [9], [18].

In this study we have conducted a further screen on the same progressing lymphomas, using a deep sequencing method (splinkerette/454) which is orders of magnitude more sensitive than previous shotgun cloning methods. Sequencing at this depth raises another potential concern, as γ-retroviruses including MoMLV display preferential integration at transcriptional start sites and other chromatin feature that may also entail a bias towards proto-oncogenes [19]–[21]. However, we present multiple lines of evidence for post-integration selection as the dominant force shaping the progression ‘integrome’. Moreover, we find that a surprisingly large fraction of the known MoMLV target gene spectrum is detectable in the integrome, indicating that any one among hundreds of genes can contribute to driving clonal outgrowth. However, there is a clear hierarchy of target genes that are selected from a large gene pool generated by the intrinsic preferences of γ-retrovirus integration. Another striking finding is the genetic bottleneck to transformation imposed by transgenic CD2-*MYC*, which is highly dependent on *Runx* gene activation. Comparison with other transgenic models of Myc over-expression shows that these each display potent selection from a small pool of master collaborating genes. These genes share the capacity to suppress the p53 pathway but are differentially recruited according to lymphoid lineage and developmental stage. The identification of a small gene set that confers the lymphoma initiating cell phenotype and is conserved in human disease has significant implications for targeted interventions.

## Results

### Deep sequencing of progressing lymphomas reveals a Darwinian clonal selection process involving many target genes

**Figure 1:**
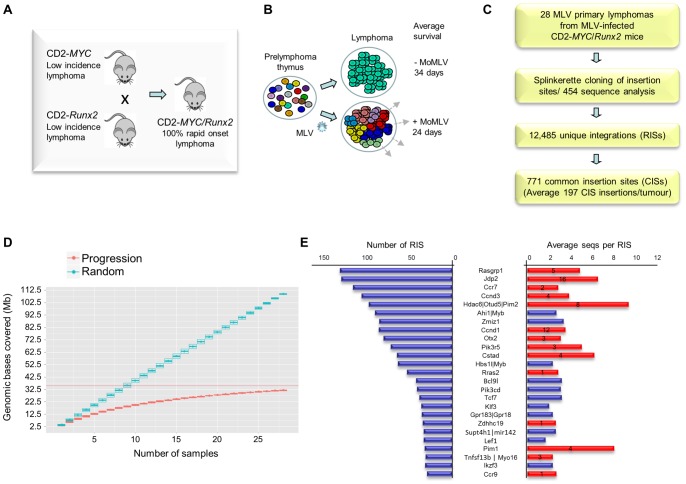
(A) Features of the system and experimental design of the RIM/DS progression screen (see also Figure S1). Mice carrying CD2-*MYC* or CD2-*Runx2* transgenes each develop a low incidence of lymphoma, while double transgenics develop lymphomas early with 100% penetrance. (**B**) Infection of double transgenic mice with MoMLV increases the rate of lymphoma development and the clonal complexity of the resulting tumours. (**C**) Flowchart of sequencing analysis: Splinkerette clones from 28 double transgenic tumours were sequenced by Roche 454 to identify 12,485 unique retroviral insertion sites (RISs). Gaussian kernel convolution statistical analysis identified 771 common insertion sites (CISs). (**D**) Saturation analysis of common insertion sites from the 28 MoMLV-accelerated thymic lymphomas. The number of genomic bases covered by predicted CISs increases as the number of samples used increases. The increase is linear if RIS are randomly distributed (upper) but approaches saturation in our real dataset (lower), indicating that 28 samples is sufficient to identify almost all positively selected CISs in this experimental system. (**E**) The 25 most frequently targeted CISs ranked by number of individual RISs. The right-hand panel shows the average number of reads for RIS. Red bars denote those detected in a previous shotgun cloning screen, with numbers denoting the number of clones detected [9]. A positive correlation (R = 0.56) was noted between with the number of reads/RIS and likelihood of detection by the lower-powered shotgun cloning methodology.

Relevant features of the CD2-*MYC* and CD2-*Runx2* transgenic mice are displayed in Figures 1 and S1. The disease-free survival of most parental transgenic mice has been attributed to variegated expression under CD2 locus control region (LCR) control [22] along with counter-selection by failsafe processes [22], [23]. As previously described [9], [17], [23], co-expression of both transgenes results in rapid onset lymphomas in 100% of mice, but the tumours typically display a single predominant clone as illustrated by T-cell receptor gene rearrangement (Figure S1). Neonatal infection with MoMLV leads to accelerated lymphoma onset, increased clonal complexity and lymphoid dissemination, although the tumours retain the characteristic bimodal phenotype seen in the absence of infection (CD8+,CD4+/−,TCRhi) [16].

Here, a panel of 28 lymphomas was analysed by RIM/DS (splinkerette/454). Processing of reads as described in Methods yielded 12,485 unique retroviral insertion sites (RISs), compared to 272 by previous manual cloning and sequencing methods [9]. Common insertion sites (CISs) were identified using a multi-scale Gaussian Kernel Convolution approach [24] yielding 771 significant CISs compared to 0–3 expected from simulations of random integration (Table S1). A list of all RIS is provided as a .bed file for visualisation in genome browsers, version mm9 (Table S2). Notably, analysis of CIS accrual by number of tumours indicated that this system is approaching saturation and that virtually all the retrievable CISs have been detected (Figure 1D). Target genes affected by integration at CISs were identified by computational methods [25] followed by manual curation.

All 14 target genes identified by shotgun cloning methods [26] featured prominently (Figure 1E; Table S3). There was a positive correlation between the number of clones previously detected by shot-gun cloning and the number of 454 reads (linear regression analysis; R = 0.56) showing that earlier lower powered methods detect only the “tip of the iceberg” of clonal expansion. While splinkerette/454 analysis is only semi-quantitative due to restriction enzyme site distribution and primary sequence constraints on PCR efficiency, we noted that the most abundant RIS corresponding to *Pim-1* insertions were also detectable as rearrangements by Southern blot analysis (Figure S2). Moreover, the top 40 RISs (by number of reads) show few apparent passenger insertions, defined as isolated RIS far from any known target gene (5/40), although these predominate (85%) in the total population of 12,485 RISs. The possibility that most of these clones have acquired two separate driver insertions without any passenger insertions appears unlikely, suggesting that most highly proliferative clones contained only a single provirus.

### Comparison with CISs from end-stage MoMLV lymphomas reveals major overlap

**Figure 2:**
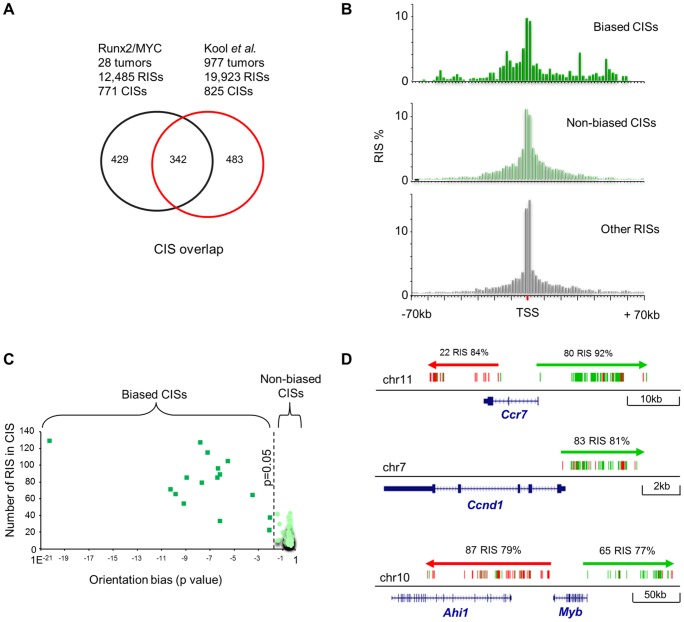
(A) Comparison between CISs detected in 19,900 MoMLV insertions derived from 937 lymphomas by shotgun cloning [25] and the progression CISs defined in this study. There is substantial overlap in the CISs detected. (**B**) Peak distance of RISs from the nearest transcription start site (TSS). RISs that fall outside CISs (bottom panel) display a distribution similar to that reported for unselected MoMLV insertions [19], [21], and strongly cluster around the TSS. RISs that comprise the ‘biased CIS’ set (top panel) display a relaxed clustering at TSS, while ‘non-biased CIS’ set present an intermediate picture. (**C**) Orientation bias analysis of 771 progression CISs. Bias is plotted against the number of RISs in each CIS (after Benjamini-Hochberg correction for multiple testing). Those with a p-value <0.05 define the ‘biased CIS’ set, and the others the ‘non-biased CIS’ set. (**D**) Examples of orientation bias of RISs targeting *Ccr7*, *Ccnd1* and *Ahi1/Myb*. Each vertical bar represents an individual RIS, coloured to depict orientation (green forward, red reverse) relative to the DNA+strand. Positions of exons and introns are abstracted from the UCSC genome browser (NCBI37/mm9). Percentages refer to predominant orientation at each CIS. Notably, this analysis implicates *Myb* as the target of long-range insertions from both 5′ and 3′ ends.

If the progression network consists of target genes that can complete the oncogenic transformation process, they would be expected to feature strongly in the dominant clones found in end-stage MoMLV-induced lymphomas. To test this assumption, we examined the overlap between the 771 progression CISs in this study with a meta-analysis by Kool and co-workers involving CISs identified by shotgun cloning of 19,923 unique RIS from 977 MoMLV-induced lymphomas of wild-type or tumour suppressor deficient mice [3]. Due to the lower sensitivity of the approach, these CISs should be enriched for major expanded tumour clones. A remarkable 346 CISs (45%) were found in common between the Kool CISs and the progression CISs, indicating that a significant proportion of the target genes involved have been implicated previously as drivers of lymphoma development (Figure 2A, Table S4).

### Further evidence of oncogenic selection: orientation bias and network analysis

Preferential integration of γ-retroviruses around transcriptional initiation sites is an established phenomenon [19] and on the basis of this and further evidence of non-random behaviour it has been argued that the observation of a CIS is insufficient evidence that post-integration selection for growth has occurred, particularly in large scale analyses [27]. While the ideal comparison with the progression CISs identified here would be normal thymocytes immediately after infection, there are significant technical challenges in obtaining a reliable *in vivo* baseline measurement due to the kinetics of infection and ongoing replication. We therefore chose to compare some aspects of our data to a published large-scale study of human CD34^+^ cells obtained after *in vitro* infection with a non-replicating MLV vector. This study by Cattoglio *et al.* is described as ‘near-baseline’, as analysis was not carried out until 10 days post-transduction [21].

Notably, preference for transcriptional start sites was relaxed in the CISs observed in our study and this trend was more evident still in CISs with an orientation bias, consistent with the increasing importance of post-integration oncogenic selection in this subset (Figure 2B). Moreover, we noted that most of the highly targeted CISs displayed the pronounced orientation bias that is classically associated with enhancer-mode gene activation [1]. As orientation bias does not arise at the level of integration [28], this feature provides direct evidence of post-integration clonal selection. Stringent filtering of CISs for orientation bias yielded 17 examples which we will refer to as biased CISs (Figure 2C; Table S5). We applied the same approach to the Cattoglio ‘near-baseline’ dataset [21] and found no clusters with significant orientation bias after correction for multiple testing. CIS target genes displaying strong orientation bias were also the most frequently targeted and often displayed the greatest levels of clonal expansion, suggesting that enhancer mode activation is the most efficient process by which MoMLV drives lymphoma progression.

An interesting outcome of this analysis shown in Figure 2D is that it provides strong support for the *Myb* gene as the target of long-range activation by insertions both 5′ and 3′, including the CIS annotated as *Ahi1*, in accord with hypotheses based on gene expression studies in lymphoma cell lines [29], [30]. Further examples of genes subject to enhancer mode insertions are shown in Figures 2D and S3.

Evidence that the biased CIS targets form part of a larger progression network under selection was provided by KEGG pathway analysis which showed that some of the most frequent CIS targets (e.g. *Ccnd3, Ccr7, Pik3cd, Pik3r5, Rasgrp1*) map to metanodes that include many of the less frequent targets (Figure S4). Furthermore, KEGG pathway enrichment analysis showed that statistically significant over-representation of specific signalling pathways (T-cell receptor, chemokine, JAK-STAT) was evident even when the top 50–100 target genes were excluded from the analysis (P = <1×10E-5), arguing that oncogenic selection may also be occurring at sites that harbour only a few insertions (Figure S5).

While orientation bias is useful to identify oncogenic selection on a background of preferential integration, we noted that there was a second frequent CIS group defined by intragenic insertions that displayed no statistical bias in orientation. Evidence that these are also under oncogenic selection is provided by the fact that 17 of the 20 most frequent targets have been observed in end-stage lymphomas (Table S6) and by the fact that a significant subset have annotation suggestive of tumour suppressor or oncogene function (*Ikzf3, Mad1l1, Als2, Ppp1r16b, Prex1, Ttc28 and Ptprc*). The typical pattern of insertions distributed across the target genes is suggestive of a tumour suppressor role, although a role for oncogenic truncated isoforms is also plausible [1], [9], [31]

### The progression network provides strong evidence of complementation

Although the majority of top ranking MLV target genes were shared between our progression dataset and the Kool meta-analysis of end-stage lymphomas, there were also notable differences. This was evident from comparison of CIS peak heights and relative rank order of CISs between the datasets where the most discordant examples are listed (Table S7). Oncogenic complementation was evident, with greatly reduced targeting of *Myc/Pvt1*, *Mycn* and *Runx* family genes in the progression set. However, there was also a marked loss of selection for some major targets recorded by Kool *et al.* including *Gfi1* and *Notch1*. It appears that the combination of *MYC* and *Runx2* in this context also renders these insertions redundant, which is intriguing as insertions at *Gfi1* have been shown to be positively selected in some CD2-Runx2 lymphomas [18].

Also of interest was the large number of novel CISs in the progression set (Table S8, examples shown in Figure S3). The most frequently targeted CIS targets displaying strong evidence of enhancer mode activation included *Otx2*, a homeobox transcription factor which plays a major oncogenic role in medulloblastoma [32] but has not previously been observed in haematopoietic cancers and *Myo16*, an atypical nuclear myosin with links to survival, cell cycle progression and PI3K signalling [33]. Moreover, a number of prominent targets for potentially disruptive intragenic insertions were unique to the progression set. These included *Endou* (Pp11), a placental poly-U endonuclease over-expressed in ovarian adenocarcinomas [34], *Xrra1*, which has been shown to modulate the response to X-ray irradiation [35], and *Ttc28* (Tprbk), encoding a large tetratricopeptide domain protein that is regulated by p53, complexes with BRCA1 and suppresses the growth of Ras-transformed cells [36].

### The transcriptome of prelymphoma *MYC/Runx2* thymus provides insights into progression gene selection and chemokine-receptor interplay in lymphoma dissemination

**Figure 3:**
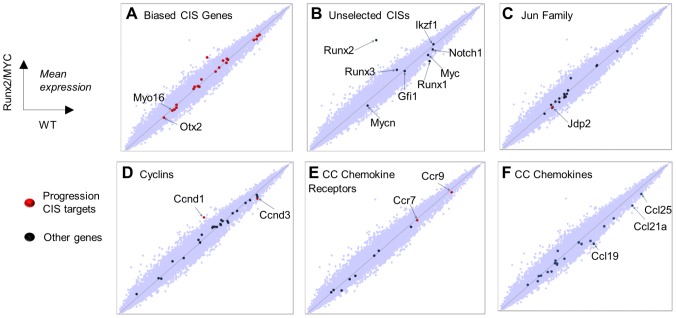
Global gene expression analysis in 10 day old (prelymphoma) *MYC/Runx2* thymus compared to wild type controls as determined by Affymetrix microarray. Scatter plots showing relative expression of genes in transgenic vs control mouse thymus with particular gene sets highlighted as indicated. Target genes positively selected in the progression network are denoted by red dots, others by blue dots (**A**) Biased CIS gene set, with annotation of novel targets (**B**) Common MoMLV target genes absent from, or under-represented in, progression CISs. (**C**) Jun family, noting the prominent target Jdp2. (**D**) Cyclin genes, noting prominent D-cyclin targets (**E**), CC chemokine receptors, noting prominent targets Ccr7 and Ccr9, and (**F**) CC chemokine ligands showing significantly down-regulated Ccl19,21,25.

A previously published analysis of preferential integration targets in early passage CD34+ cells showed a good correlation between basal transcriptional levels and integration frequency [21]. To test whether progression RIS targets were also selected by their high transcription rates in premalignant cells, we compared the transcriptomes of *Runx2/MYC* and control thymus at 10 days of age, several weeks before clonal tumours emerge. Figure 3 shows expression scatter plots for all gene probes. Basal expression of the most prominent progression targets was widely variable, and only *Ccnd1* showed significant up-regulation compared to control thymus. Moreover, the frequent MoMLV targets that were not enriched in the progression network showed a similarly wide distribution with regard to expression levels. The exquisite selection by RIM of specific members of multigene families (e.g. *Jdp2*, D cyclins) also appeared to be poorly correlated with expression level, strengthening evidence for post-integration selection as the predominant force shaping the progression network.

Frequent targeting of *Ccr7*, and to a lesser extent *Ccr9*, is interesting in view of their central roles in mediating T-cell progenitor homing to thymus [37], [38]. Moreover, Ccr7 has been reported as a mediator of progression and homing to lymph nodes in multiple tumour types, and to stimulate survival pathways by autocrine or paracrine mechanisms [39]. The cognate ligands for Ccr7 and Ccr9 (*Ccl21a*, *Ccl19*, *Ccl25*), are highly expressed in normal thymus, but intriguingly were significantly down-regulated genes in premalignant organs (validation shown in Figure S6). The respective chemokine genes are normally expressed only in non-lymphoid elements of the thymus including epithelial cells [40]. The possibility that these genes were aberrantly activated to drive autocrine growth in the lymphoma cells was tested by direct analysis of isolated lymphoma cells (Figure S7). However, expression of the ligand genes was below detectable levels in *Runx2/MYC* or CD2-*MYC*/p53 null lymphoma cells suggesting that activation of *Ccr7/9* provides a growth advantage by a paracrine mechanism that is dependent on thymic stroma. Falling expression of ligand genes in 10-day *Runx2/MYC* thymus may be due to down-regulation or simple occlusion of non-lymphoid cells by nascent lymphoma cells, which is virtually complete at later stages (Figure S1).

### Analysis of single transgenic tumours reveals a *Myc*-directed bottleneck and collaboration between *Runx2* and *Ikzf1*

**Figure 4:**
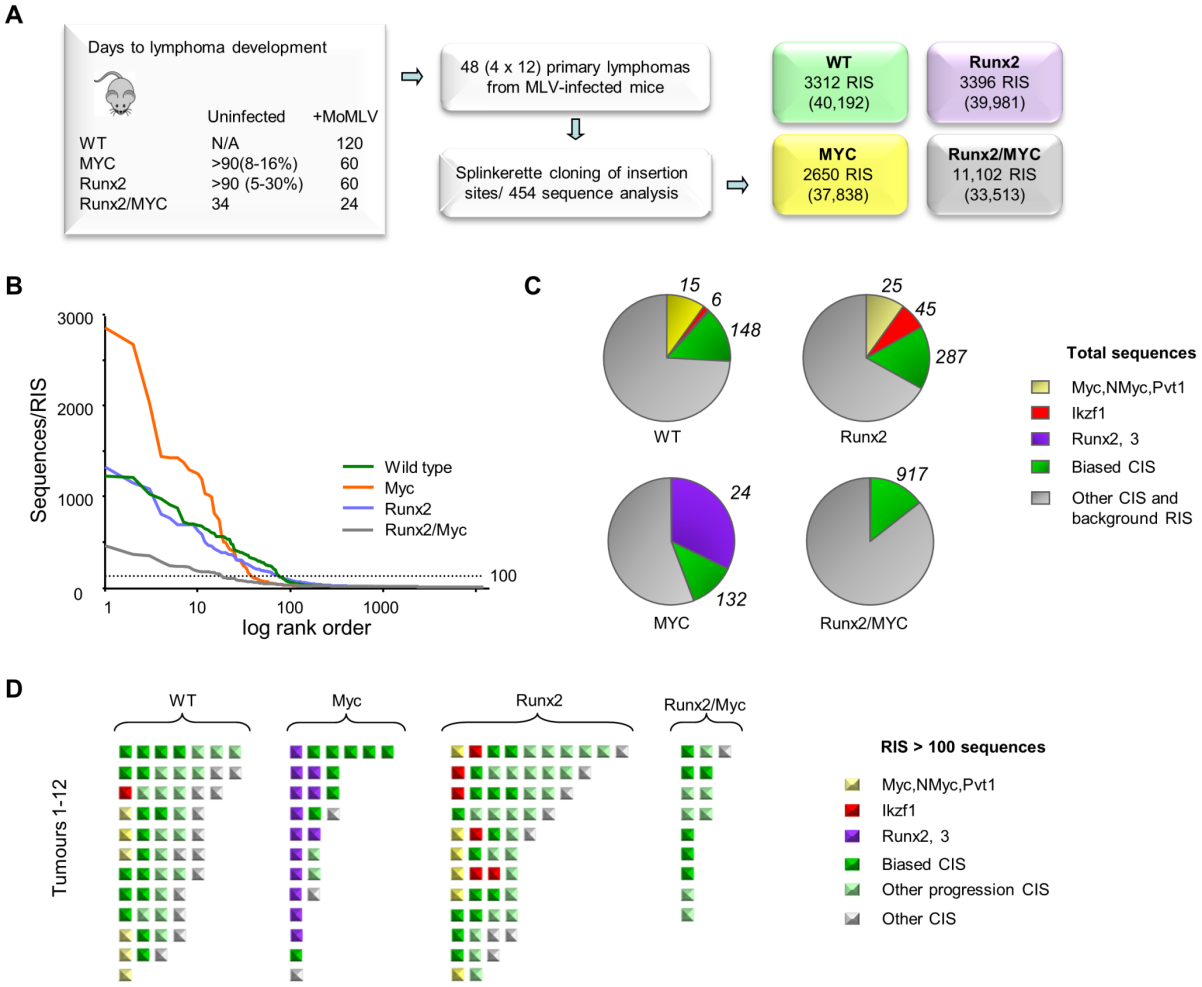
(A) Features and design of the RIM/DS complementation screen. Average lifespan (days) of wildtype (WT), CD2-*MYC*, CD2-*Runx2* and CD2-*MYC/Runx2* double transgenic mice, without and with MoMLV infection. Percentage value indicates lifetime lymphoma incidence. 12 lymphomas from each genotype were analysed by RIM/DS, identifying the indicated number of RISs (total reads in parentheses). (**B**) Individual RIS in log rank order according to number of reads. Horizontal dotted line represents 100 reads per RIS and was used as a threshold for expanded CISs shown in (d). (**C**) Total reads in each genotype cohort comprising the CISs around *Myc/Mycn/Pvt1* (yellow), *Ikzf1* (red), *Runx2/Runx3* (purple), around other CISs from the biased CIS set (green) or other RISs (grey). The total numbers of RISs that contribute to the overall read count are indicated outside the pie charts (**D**) Schematic representation of all RISs with at least 100 reads detected by DS. Each square represents a single RIS, with colour coding as in (c). Expanded RISs not falling within a CIS (presumptive passenger RIS) are not depicted. These analyses illustrate the reduced complexity and greater clonal expansion in MoMLV accelerated CD2-*MYC* tumours.

To compare the progression network with genes selected during earlier events in tumorigenesis, a second RIM/DS barcode screen was conducted, including MoMLV-infected end-stage lymphomas from parental CD2-*MYC*, *-Runx2* and wild-type mice with a subset of *Runx2*/*MYC* progressing tumours (Figure 4, Table S9). All insertions, sorted by genotype are provided as a .bed file for visualisation in genome browsers, version mm9 (Table S10). *MYC* and *Runx2* transgenes each cooperate with MoMLV to accelerate lymphoma onset to around 60 days post-infection [8], [17], [18]. Compared to *MYC/Runx2*, the other three tumour sets yielded many more reads, but from a much smaller number of unique RISs, reflecting the presence of highly expanded tumour clones (Figure 4A, B). The massive number of RISs per tumour (221–276) shows that in MoMLV lymphomas the predominant end-stage clones co-exist with a polyclonal background of minor populations.

Application of an abundance threshold of 100 copies (Figure 4B) yielded a RIS number close to that expected from Southern blot analyses of end-stage MoMLV tumours that estimated 4–6 RISs in each dominant clone [41]. In most cases this cut-off correlated well with previous direct analyses for gene rearrangement [18], [42], although rearrangements of *Myc* detected by Southern blot in two of the CD2-*Runx2* tumours analysed here failed to register in the splinkerette/454 analysis. Occasionally ‘missing’ clones might be explained by technical limitation e.g. due to sequence drift in primer sequences. In this regard, it is noted that the bias towards *Myc* family insertions was less marked here than in a Southern blot-based analysis of a larger CD2-*Runx2* tumour cohort [18]. Nevertheless, there were clear and profound differences between cohorts, as *MYC* transgenic tumours resolved into fewer clones with substantially greater clonal enrichment compared to other genotypes while the double transgenics showed greater complexity as expected (Fig. 4B, C). This apparent difference in the mode of tumour acceleration is interesting as CD2-*Runx2* mice harbour an expanded population of transformation-prone thymocytes, which has no parallel in CD2-*MYC* mice, most of which remain healthy with no obvious abnormality [8], [16], [43].

The most striking features of the single transgenic tumours were evident when the most abundant RISs were sorted according to gene family (Figure 4C, D). High copy RIS mapping to *Runx2* or *Runx3* were almost ubiquitous in, but exclusive to, CD2-*MYC* tumours (P = 0.0001, Fisher's Exact Test). A number of high abundance RIS mapped far upstream of *Runx2*, adding this gene to the list of those subject to long-range activation. Only two tumours displayed no detectable *Runx* insertion.

Another salient observation from analysis of the end-stage lymphomas was that the low abundance RIS left after subtraction of the major clones frequently correspond to progression network genes (Tables S11, S12). It is conceivable that these represent tumour subclones that have acquired a further hit of proviral insertion, although the alternative possibility that these represent insertions in prelymphoma cells cannot be excluded. The possibility that this background reflects preferential integration in untransformed cells appears unlikely, as such cells form only a tiny fraction of the thymic mass and the hallmark orientation bias at major targets (Figure 1C) is also evident in these minor populations. Moreover, expanded RIS indicative of third hit genes in CD2-*MYC*, CD2-*Runx2* and wild-type mice appeared to be selected from a broad cross-section of the progression network, with the ‘winners’ of the progression race largely recapitulating the expansion rate measured by earlier analysis of the progression network (Figure 1D).

### Second hit genes represent a narrow genetic bottleneck to transformation

**Figure 5:**
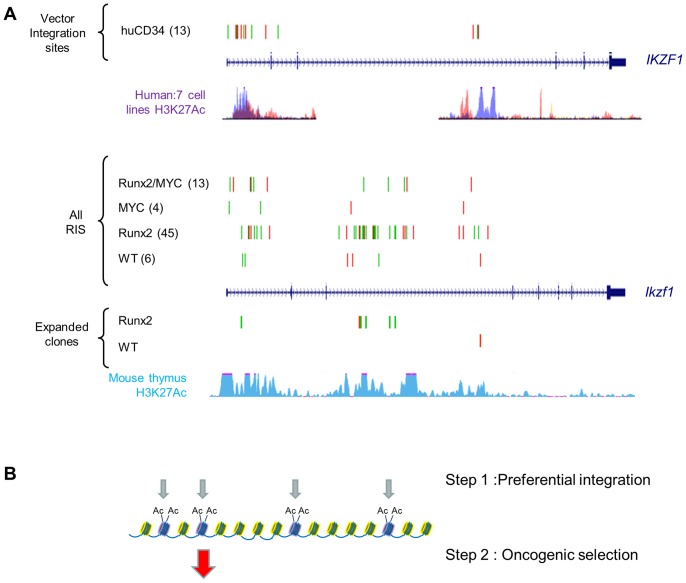
Insertions at *Ikzf1* display dual features of preferential integration and oncogenic selection. **A**) Upper panel : Inspection of >32,000 MLV vector integrations in early passage human CD34+ cells (Cattoglio set [21]) shows two clusters of integration in the *IKZF1* gene which map to sites of active chromatin marks (H3K27 acetylation, ENCODE data for 7 cell lines). Lower panel: A pattern of low abundance integrations within *Ikzf1* is present in all 4 genotypes in our study, suggesting a conserved process of preferential integration at the murine gene corresponding again to chromatin features (H3K27 acetylation in C57/BL thymus). However, many more insertions are evident in the CD2-*Runx2* background, and substantial expansions (>100 reads) in end-stage lymphomas show a similar genotype bias. (**B**) Diagrammatic model of a two-stage process of oncogenic selection on a background of MoMLV preferential integration.

We reasoned that specific ‘second hit’ collaborating genes would be distinguishable from progression genes on the basis of (a) positive selection in lymphomas of single transgenic mice compared to wild-type and (b) loss of selection or reduction to background levels in double transgenics. As expected, the *Runx* genes (*Runx2* and *Runx3*) and *Myc* family targets (*Myc*, *Mycn*) conformed to this pattern, being selected in CD2-*MYC* and CD2-*Runx2* respectively and effectively disappearing from the double transgenic tumours (Figure 4C). Surprisingly, inspection of the entire CIS list revealed only one other target gene with statistically significant correspondence to this pattern: intragenic insertions in *Ikzf1* were significantly more abundant in CD2-*Runx2* transgenic tumours than in the other three genotypes and showed more frequent representation in dominant clones (Figure 5). Intriguingly, analysis of the CD34+ ‘random integration’ vector dataset [21] shows two hotspots for integration in the human *IKZF1* gene that correspond to active chromatin marks. The murine *Ikzf1* gene showed a similar background pattern, although 3–4 clusters of insertions could be discerned in the murine gene. These observations suggest a two-step model for targeting of *Ikzf1* by MoMLV, with preferential integration at sensitive sites within the gene leading to sustained clonal expansion only in the presence of a collaborating lesion such as deregulated *Runx* expression (Figure 5B).

**Figure 6:**
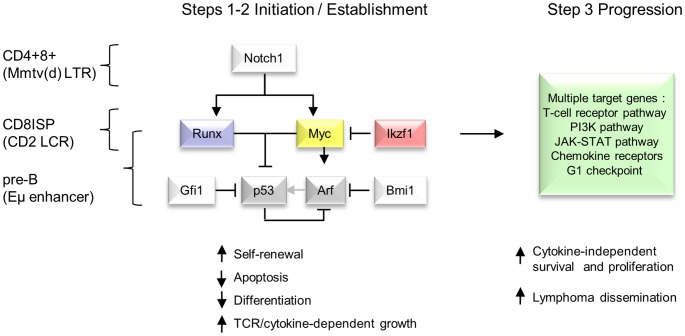
Hierarchical model of MoMLV-induced T-cell lymphomagenesis and preferred target genes in Myc transgenic systems. In this model, critical genes in lymphoma initiation and establishment converge on *Myc* and p53, while *Ikzf1* insertions are postulated to de-repress c-*myc* [31], [67]. Lymphoma phenotype and preferred target genes vary according to expression control element and lineage [7], [53], [56]. A much larger set of target genes and downstream pathways is capable of driving clonal expansion at the tumour progression stage.

Table S13 summarises the genes showing strongest evidence of complementation in parental transgenic mice. In addition, there is evidence of reduced selection for *Gfi1* and *Notch1* insertions on the CD2-*MYC* background which directly mirrors findings on the progression set compared to common MoMLV targets (Table S7) suggesting that this bias is conferred by the CD2-*MYC* transgene. Targeting of both genes in wild-type controls and CD2-*Runx2* in this study rules out mouse strain differences as the basis of this phenomenon. Notably, Notch1 has been shown to block p53-dependent apoptosis due to Myc over-expression [44], while Gfi1 has recently been shown to modulate p53 responses indirectly by altering protein methylation [45]. The latter finding illuminates early RIM screens of Eμ-*Myc* mice which suggested that *Gfi1* and *Bmi1* belong to the same complementation group [7], and Bmi1 is known to control p53 responses by transcriptional suppression of *Arf* [46]. As we have shown that Runx2 also inhibits Myc-induced apoptosis *in vivo* and that the *Runx2/MYC* combination neutralises selection for loss of p53 [16], we propose the model in Figure 6 to account for the respective gene interactions in different transgenic backgrounds in a three-hit model of MoMLV lymphomagenesis.

### Comparison with murine and human T-cell lymphomas reveals extensive overlap in common insertion sites and domains of copy number alteration

The extent to which the pathways targeted in retrovirus-induced lymphomas overlap with similar tumours of non-viral origin, including human cancers, is also of considerable interest. We compared the comprehensive CIS database with regions of chromosomal gain and loss described in a previous study of T-cell lymphomas arising in mice defective in telomerase, p53 and ATM (‘TKO’) mice [47], where a strong overlap was noted with human T-ALL. Remarkably, 16/18 regions of syntenic deletion or amplification contained CISs, corresponding to 43/771 CISs (for this overlap P = <0.0001; Table S14). Notably, no known cancer genes could be found at the majority of these domains [47], suggesting that the genes targeted at these CISs represent candidates for gain or loss of function that is conserved between human and mouse cancers. Significantly, many of the target genes display intragenic insertions, particularly for the deleted regions (13/22). An interesting example is Gpr132, located on chromosome 12, which encodes a G-protein coupled receptor with apparent tumour suppressor activity [48].

## Discussion

In this study we examined an established system of oncogene cooperation and retroviral acceleration using a deep sequencing (DS) platform. RIM/DS increases sensitivity of RIS detection by almost two orders of magnitude over earlier methodologies [9] and when applied to a lymphoma progression model shows evidence of saturation, indicating that all relevant major CISs have been obtained. The remarkable observation that much of the large repertoire of MoMLV target genes from almost one thousand end-stage T-cell lymphomas can be found in the progression network from only 28 lymphomas shows the enormous potential of RIM/DS when applied to polyclonal populations under strong selection. While statistical and pathway analyses provide useful tools to discriminate genes under oncogenic selection from preferential integration, our findings suggest that the phenomena may not be completely separable. The example of *Ikzf1* illustrates the principle whereby a gene may be selectively targeted by γ-retroviral integration but leads to clonal expansion in the presence of a complementary oncogenic programme provided in this case by Runx gene activation. It has been demonstrated recently that γ-retroviral integration at transcriptional start sites is a consequence of interaction with BET chromatin regulators that direct the process towards genomic regions rich in acetylated histones [49], [50]. The integration specificity of γ-retroviruses is clearly fundamental to their efficient replication and transmission in nature. In wild-type mice, the rate of oncogenic transformation due to successive integration events is reduced by retroviral interference, but the process is accelerated in oncogene transgenic mice where fewer hits are required.

The implications of our analyses are also interesting for retroviral vector-based gene therapy. As the most potently selected insertions mediate enhancer-mode gene activation, the removal of enhancer elements in self-inactivating vectors [51] is likely to improve safety margins. However, failure to deal with the targeting apparatus will leave a residual risk, particularly for gene disruption events which, from their lack of obvious orientation bias, may not require strong enhancer function (e.g. at *Ikzf1*).

While intrinsic preference for integration at transcriptional start sites and other chromatin features [19]–[21] creates the platform on which oncogenic selection operates, it is clear that post-integration selection events play a decisive role in shaping the genetic profile of end-stage tumours. The progression network is highly adapted to the T-cell environment but is not simply a cross-section of highly expressed and therefore available target genes. This principle is illustrated by the strong selection for specific members of multigene families (e.g. Jdp2, D cyclins) that show no correlation with basal transcription levels. Similarly the targeting of novel genes that were not seen in previous large-scale screens of MoMLV-induced T-cell lymphomas (e.g. *Otx2*, *Myo16*) is not merely due to their up-regulation in the background of the *Runx2/MYC* model. These findings suggest that it will be of value to employ RIM/DS to probe the growth checkpoint networks in tissues and cell lineages that have been less well explored to date.

While most of the functionally annotated progression network genes are predicted to confer autonomous proliferation, an exception to this rule was provided by the frequent activation of *Ccr7* and *Ccr9*, which in their normal developmental roles promote T-cell homing to thymus and ligand-dependent survival and proliferation [37]. Moreover, *Ccr7* is stimulated by Notch signalling [52], and we would predict that retroviral activation bypasses this requirement. It appears that the result of Ccr7/9 activation in Runx2/MYC lymphomas is likely to be paracrine growth stimulation, as expression of the cognate ligands (Ccl19, 21, 25) is restricted to thymic stromal cells. Moreover, declining levels of ligand transcripts in Runx2/MYC thymus offers a rationale for the accelerated dissemination of lymphoma cells towards highly expressing peripheral lymphoid tissues [9]. Export of lymphoma cells with *Ccr7* insertions is also in accord with the relatively low read/RIS ratio in primary thymic lymphomas. Identification of *Ccr7* as a major target highlights the complementary value of RIM screening, as this gene does not appear to be subject to mutation or amplification in human cancer, yet is required for CNS metastasis of human leukaemia cells [52].

Comparison of the progression network with a large scale meta-analysis of MoMLV targets in T-cell lymphomas from various genetic backgrounds [3] showed that the principles of complementation apply where the two germ-line oncogenes are present, as insertions at *Myc* and *Runx* family members were massively under-represented in the progression set. Moreover, while most major targets overlapped strongly, a few prominent targets including *Gfi1* and *Notch1* were also greatly diminished in the progression network. Our second RIM/DS of parental transgenic mice shed further light on this observation, as the CD2-*MYC* parental transgenic system in particular did not select for these targets but instead showed virtual dependence on activation of a *Runx* family gene with the order *Runx2>Runx3>Runx1* in targeting frequency in accord with previous observations [53]–[55]. Comparison of several Myc transgenic model systems (CD2-*MYC*, Eμ-*Myc*, Mmtv(d)-*Myc*) shows that these have massively divergent preferences for collaborating genes detected by RIM, presumably reflecting the lineage and stage-specificity of *Myc* expression control [7], [42], [56]. However, it is notable that all of these potently selected collaborating genes share the ability to suppress the p53 response in the context of activated Myc [16], [44], [46], [57]. There is an obvious parallel with the observation that the combination of CD2-Runx2/MYC overcomes the requirement for genetic inactivation of the p53 pathway [16], providing a rationale for the reduced selection for Notch and Gfi1 on this background.

The foregoing observations invite the model presented in Figure 6, where the interaction of this small gene set is presented as a bottleneck to transformation in contrast to the broad range of progression genes that can be recruited at later stages. In addition to the simple outline shown here, it appears that the MoMLV ‘core’ gene programme can also neutralise p53-independent failsafe pathways, as p53 deficiency has relatively modest effects on MoMLV-induced tumour onset and target gene spectrum [5], [14], [15]. It should also be noted that at least some of the genes in the progression network can also serve as initiators when expressed as transgenes, showing that the mutational order may not be fixed [58]–[60].

Why do the major collaborating gene targets vary so markedly between Myc transgenic models? The most obvious rationale is presented by the lineage and stage-specificity of Myc expression. RIM targeting of *Bmi1* is largely a feature of B-cell lymphomas in the mouse [7], while Notch targeting predominates in the CD4+CD8+ lymphomas of Mmtv(d)-Myc mice [56]. The CD2 LCR confers strong T-cell specificity but is also active in B-cells [61], implying that its developmental activation may occur at the level of committed lymphoid progenitors. High level Myc expression in this niche appears to lead to cell death, unless combined with loss of p53 or an activated *Runx* allele [17], [62], [63]. We hypothesise that *Notch1* or *Gfi1* pathways are not available for RIM targeting at this stage and that *Runx2*, the ‘bone-specific’ family member, which is also transcriptionally active in early haematopoietic development [64], becomes the primary target for activation in this niche. As mounting evidence indicates that *Runx* family members are downstream of Notch signalling in expression control and effector functions [65], it is tempting to suggest that dual activation of *Runx* and *Myc* supplants the need for activation of Notch. The model we propose has implications for therapeutic targeting of Notch signalling with γ-secretase inhibitors [66], as up-regulation of *Runx* and *Myc* may represent another pathway to resistance.

Although CD2-*Runx2* selects strongly for activation of *Myc* family genes by RIM [18] it appears less critically dependent, possibly due to the survival of Runx2 expressing thymocytes as a premalignant, slowly proliferating population blocked at the DN/CD8ISP stage [43]. This study shows that *Ikzf1* is also favoured as a collaborating target on this background. Notably, *Ikzf1* is a haplo-insufficient tumour suppressor that has been reported to act as a transcriptional suppressor of Myc [67], while intragenic retroviral insertions lead to expression of truncated isoforms with dominant negative potential [31]. We therefore suggest that de-repression of Myc may be one of the consequences of *Ikzf1* targeting that leads to its co-selection with *Runx2*. It would interesting in this regard to test whether lymphomas of *Runx2* transgenic mice with reduced *Ikzf1* function [68] would show reduced RIM targeting of both *Myc* family genes and *Ikzf1*.

This analysis has wider implications for the genetics of human lymphomas and other cancers. It appears that the final step in lymphoid transformation by MLV can be accomplished by a wide range of genes with the common functional end-point of growth factor-independent proliferation. As the progression network also includes numerous genes that are mutated, amplified or deleted in human cancer (Table S14), it is tempting to suggest that many of the acquired mutations in human cancer are also late embellishments. Another important insight is provided by the evidence of a small network of genes (*Myc, Runx, Ikzf1, Gfi1, Notch1,* and *Bmi1*) that act in pairwise combinations to confer lymphoma self-renewal and overcome failsafe responses via the p53 pathway. It seems likely that this network operates under normal physiological conditions to licence cell growth and is co-ordinately subverted in cells carrying mutations in the pathways. The recent description of Gfi1 as an ‘oncorequisite’ factor that is rarely directly mutated but nevertheless required for growth of ALL cells [45] highlights the potential for targeting this network. The Runx genes are heavily implicated in human leukaemia but show paradoxical features of either gain or loss of function in disease subsets [69]. The demonstration here that Runx activation is virtually essential for MYC transformation of early murine T-cell lymphoma suggests that it may be fruitful to examine the requirement for RUNX function in human leukaemia/lymphomas driven by amplified MYC or NOTCH/IKZF1 mutations.

## Methods

### Ethics statement

Animals were routinely monitored and sacrificed when showing signs of ill health in line with the UK Animals (Scientific Procedures) Act, 1986.

### Animals

CD2-*MYC*, CD2-*Runx2*, and CD2-*MYC*/CD2-*Runx2* transgenic animals and maintenance were described previously [9]. Neonates were infected within 24 hours of birth with ∼10^5^ infectious units of MoMLV as previously described [42]. Littermate-matched genotype controls were used to control for mouse strain.

### DNA extraction

DNA was extracted from approximately 20 mg of frozen enlarged lymphoid/tumour tissue using Gentra Puregene Genomic DNA Purification Kit (Qiagen, UK) according to the manufacturer's instructions.

### Isolation of retroviral insertion sites

Isolation of the retroviral insertion sites from the tissues was performed using splinkerette PCR to produce barcoded PCR products that were pooled and sequenced on 454 GS-FLX sequencers (Roche Diagnostics platform) as described previously [70], [71]. The restriction enzymes used to digest the genomic DNA were Sau3AI and Tsp509I, and the enzyme used to digest MoMLV DNA was EcoRV.

### Bioinformatic analysis of 454 sequencing results

Processing of 454 reads, identification of insertion sites, and Gaussian kernel convolution (GKC) statistical methods used to identify common insertion sites (CISs) have been described previously [6], [24], [71], [72]. In summary, 454 reads were mapped to the mouse mm9 genome assembly, where the only modification to the previous alignment procedure was the removal of the stringency check as to whether an alignment was located neighbouring a TA dinucleotide site (the insertion locations preferred by *Sleeping Beauty* transposons on which the bioinformatics processing method was developed). Reads from the same sample whose start genomic locations aligned within three nucleotides of each other were merged together. Reads from the same sample that were more than three nucleotides apart were considered independent integration events. CISs were identified using the multi-scale GKC approach [6], [24]

### Analysis of sample saturation

In order to determine whether the MLV screen had reached some level of saturation, the Gaussian Kernel Convolution (GKC) CIS calls from all 28 samples were analysed using the ACT software package [73].

ACT considers genomic locations generated by multiple samples for specific biological phenomenon under study (e.g. ChIP-seq peaks) to determine the saturation of a screen. The program considers the various combinations in which samples can be added so that the increase in base pair coverage is a range of values based on all the samples. The results can be depicted as a series of boxplots showing the increase in base pair coverage, where the boxplot at each position n on the x-axis shows the coverage values of all combinations of n samples. Boxplots that approach a horizontal asymptote indicate that the coverage has reached saturation.

For the GKC CISs generated by all 28 samples, the insertion sites that contributed to CISs were extracted, resulting in a set of 7,485 sites. The insertion sites were then selected per sample and pseudo-kernels of 7.5k nucleotides either side of each insertion were applied to mimic GKC kernels of 15k nucleotides. Overlapping kernels within each sample were merged into continuous genomic regions. These 28 modified insertions files were then analysed using ACT. For each combination of samples the median values, and 25th and 75th percentiles were plotted using ggplot2 [74].

As a control, the 28 samples were re-analysed where the same number of insertion sites per sample were selected at random across the mouse genome. The pseudo-15k nucleotide kernels were applied.

While the analysis does not produce a clear-cut asymptote this is to be expected due to the type of data under consideration. ACT was designed to analyse such data as ChIP-seq arrays for predicting transcription factor binding sites. In these scenarios ChIP-seq replicates should ideally report the same key binding sites/genomic locations. Hence across multiple samples the same locations should be reported.

For MLV screens however, while insertions in the same gene will be found from different samples, the locations of the insertion sites will not overlap perfectly, even with the addition of the 15k nucleotide pseudo kernels. Hence each sample will introduce novel regions, such that the overall coverage will continue to increase even if the screen has truly reached a ‘saturation’ point. Also not all samples will contribute to all CISs. Different combinations of samples will thereby result in varying coverages, causing the coverage profile not to asymptote perfectly.

### Integration site location mapping relative to transcription start sites (TSS)

The genomic coordinates of the ‘UCSC Genes’ set was downloaded via the UCSC genome browser for mouse assembly mm9. Each of the 12,485 MoMLV integration sites was then mapped relative to the transcription start site (TSS) of its closest UCSC-defined ‘known’ gene.

### Bioinformatic analysis of Kool et al. 2012 insertion sites

The Kool set of 19,923 mouse retroviral insertions sites was downloaded from the Mutapedia website (http://mutapedia.nki.nl/) [3]. In the original paper, 596 CISs were identified using the GKC statistical framework with a fixed kernel width of 30k nucleotides. The insertion sites were re-analysed using the same multi-scale kernel approach that was applied to the MoMLV insertion sites. As a result of the multi-scale kernels and a less stringent cut-off value, 977 CISs were identified.

Defining the width of a CIS as spanning the minimum and maximum genomic coordinates of insertion sites that contribute to a CIS, CISs were compared between the progression set and the re-analysed Kool set for overlaps. CISs were called overlapping if at least one nucleotide was overlapping between the two CIS sets.

### Integration site orientation bias analysis

#### MLV CISs from this study

For each MoMLV CIS, the integration sites that contributed to it were collated, divided into forward- and reverse-orientation sites, and their frequencies counted. A one-tailed Fisher's exact test was then performed using the frequencies of the CIS-specific integrations versus the frequencies of remaining integration sites not present in the current CIS. Multiple test correction was performed using the Benjamini-Hochberg procedure [75].

### MLV vector CISs in CD34+ cells

A set of 32,592 human MLV-based vector integration sites was kindly provided by Cattoglio and co-workers as previously published [21]. In the original study genomic regions were considered as significant if three or more integration sites were found clustered within regions of 12,587 nucleotides. This threshold was applied to the 32,592 integrations sites resulting in the identification of 3,453 clusters. Taking the integration sites within the clusters, a similar Fisher's exact test method was used to assess the orientation bias of the integration sites as for the MoMLV CISs. Following multiple test correction no clusters exhibited any orientation bias.

### Microarray analysis

RNA was isolated and purified from the thymuses of 10 day old wild type and CD2-*MYC/Runx2* double transgenic mice using an RNeasy Mini Kit as per the manufacturer's instructions (Qiagen, UK) with mechanical lysis using a pellet pestle in a microfuge tube (Sigma). RNA purity was assessed using a Nanodrop 2000 Spectrophotometer (Thermo Scientific), and integrity verified using the Agilent 2100 Bioanalyser with RNA 6000 Nano Reagents kit (Agilent Biotechnologies) as per the manufacturer's protocol. Whole genome expression profiling was performed using Affymetrix mouse GeneChip microarrays (MoGene-1) in triplicate as per the manufacturer's protocol (Affymetrix, UK). Data analysis was carried out using the Partek Genomic Suite (Partek Inc., St. Louis, MO, USA). Briefly, after Robust Multichip Average normalisation [76] with GC content pre-background adjustment, the differentially expression analysis was performed using ANOVA. Multiple testing correction was done using the ‘q value’ cut-off [77] with gene changes of p<0.05 considered significant. Graphical representations of data were prepared using CLC Genomics Workbench 4.

## Supporting Information

Figure S1 (**a**) Basic features of the lymphoma model. Expression of either *Runx2* or *MYC* under the control of the CD2 locus control region leads to a low lifetime incidence of T-cell lymphoma. This appears to be due to the variegated activation of the transgenes and counter-selection for expressing cells which either die by apoptosis (MYC) or grow slowly with impaired differentiation (*Runx2*). The combination of both transgenes cancels these failsafe responses and leads to early onset lymphoma in a 100% of mice [8], [17], [18], [23]. Tumour onset can be accelerated further by neonatal infection with Moloney murine leukaemia virus (MoMLV) [26]. (**b**) The clonal nature of CD2-Runx2/MYC lymphomas is demonstrated by rearrangements of the T-cell receptor β-chain. Southern blot analysis of 20 mg samples of DNA digested with HindIII and analysed with a Cβ probe. The virtual disappearance of the unrearranged Cβ1 is due to the replacement of non-lymphoid cells by lymphoid cells carrying deletions or rearrangements of Cβ1. As TCR rearrangement can result in productive rearrangement or deletion of Cβ1, dominant clones may be represented by one or two bands. As can be seen, spontaneous tumours in these mice typically display a single major clone, although some evidence of minor clones is present in some cases (-MoMLV). In MoMLV accelerated tumours, there is typically a more complex pattern indicative of greater clonal complexity. Due to the limited sensitivity of Southern blot analyses, clones representing less than 5% of the tumour mass are not detectable. **c** Phenotypic analysis of CD4 and CD8 expression in primary thymic lymphoma CD2-Runx2/MYC mice. Note that normal thymocytes were almost completely replaced by the characteristic bi-modal tumour cell population (>96–99%). No phenotypic difference was observed in MoMLV-accelerated lymphomas.
(TIF)

Figure S2 (**a**) Evidence that expanding clones in virus-accelerated Runx2/MYC lymphomas contain a single provirus. The top 40 RISs (in rank order by number of reads) shows few insertions at isolated RIS far from known target genes(5/40), although these predominate (85%) in the total population of 12,485 RISs. If clonal expansion required two or more hits of proviral insertion, we would expect many more instances of co-amplification of passenger RIS (grey bars). (**b**) There is a correlation between splinkerette 454 sequence reads and Southern blot detection of rearrangement, with insertions at Pim1 in expanded tumours clones in tumours 20i and 13i being detected by both methods at similar relative efficiency (compare to (a)). G: germ line; R: retrovirus insertion.
(TIF)

Figure S3 Additional MLV insertion patterns at other biased and non-biased CISs. Each vertical bar represents an individual RIS, red indicates reverse orientation compared to the+strand, green the same orientation. The positions of exons and introns were extracted from the UCSC genome browser (NCBI37/mm9).
(TIF)

Figure S4 KEGG Cytoscape plot. Genes with RIS counts of 3 or more are visualized in the context of their KEGG pathway interactions using Cytoscape. The KEGG network is based on metanodes. A metanode is a collection of genes that share similar function. Some metanodes only contain a single gene. Links in the KEGG network denote a functional interaction between any of the genes in the two metanodes connected by the link. For visualization purposes the metanodes themselves are not displayed. Consequently, in the resulting graph a link between two genes is present if there is a link between the metanodes in which these genes reside. Blue links are KEGG pathway links, red links connect genes that are in the same metanode in KEGG. Genes that are in the same metanode share functionality (according to KEGG). Note that metanodes are not necessarily consistent across different pathways, which is why some genes that are in the same metanode have a different set of interaction partners. Node colour and size represents the number of RIS attributed to that gene: blue and small circle: 3 RIS, red and large circle: up to a maximum of 127 RIS.
(TIF)

Figure S5 KEGG pathway enrichment analysis. The effects on pathway analysis of limiting gene sets by number of hits or removal of most prominent CISs. This analysis was conducted to test the extent to which oncogenic selection is present throughout the detected RISs. Box plots represent log_10_ p-values for all pathways in the KEGG database. The legend lists the pathways with a significant p-value (at the 1×10E-5 level) for at least one of the discovery set definitions, with the minimum p-value between parentheses. The leftmost box depicts the log_10_ p-values of the pathway enrichment when the discovery set is defined as all genes associated with five or more RISs. For the second box from the left the discovery set is defined as all genes with at least one associated RIS. This is also the case for box 3 through 6, but in those discovery sets the top 20, 50, 100 and 500 most frequently targeted genes are removed from the discovery set, respectively. The horizontal red line indicates the 10^−5^ significance level. Surprisingly, enrichment is more significant when the entire ‘integrome’ is analysed than when restricted to genes that are frequently targeted (by 5+ RIS). Moreover, removal of 20 to 100 ‘top hit’ genes which includes genes common to many of the annotated pathways (e.g. Ccnd, PI3K, Pim gene families) has relatively modest effects on significance scores, while enrichment for pathways in cancer and others survives even the removal of the top 500 genes. These results strongly indicate that either a) the majority of RISs, including those that are not common across multiple tumours, have been subjected to oncogenic selection or b) viral targeting of these pathways is an underlying phenomenon based on integration preference.
(TIF)

Figure S6 (**a**) Quantitative real-time PCR validation of key gene changes observed in the microarray. Quantification is relative to house-keeping gene TBP for genes in the CC chemokine family, with fold changes and significance as determined by two-tailed unpaired student's T-test shown in (**b**) Error bars represent S.E.M. Genes with fold differences reaching a q<0.05 significance threshold in the microarray are noted with an asterisk (*). N/A = gene not present on the microarray.
(TIF)

Figure S7 Lack of expression of CC chemokine genes in lymphoma cells from Runx2/MYC and other genetic backgrounds. Quantitative real-time PCR analysis of CC chemokine receptors and ligands for a number of T-cell lymphoma lines from Runx2/MYC (GIM) or p53null/MYC (p/m) backgrounds, expressed relative to adult normal thymus, with HPRT as control. (**a**) Ccr7 and ligands (**b**) Ccr9 and ligand. (**c**) Significance of down-regulation of CC ligands compared to receptors in T-cell lines, determined by two-tailed unpaired student's T test. Errors represent standard error (SEM).
(TIF)

Table S1 Master list of all CISs showing CIS chromosomal location and peak height, plus associated genes. Also shown is the number of insertions in each CIS.
(XLSX)

Table S2 .bed file of all GIM1 alignments, showing chromosomal locations, tumour identity, read counts and strand polarity.
(XLSX)

Table S3 The top 25 progression CISs, ordered by the number of insertions. Gene names and annotations are shown, as are the total number of insertions, the number of tumours with insertions and the average number of reads per RIS for each gene. Genes in bold were previously identified as targets by shotgun cloning.
(TIF)

Table S4 Overlapping CISs found in both this screen and the Kool *et al.* screen, showing CIS chromosomal locations and gene identities.
(XLSX)

Table S5 CISs with significant orientation bias, showing gene identities, the total number of insertions, the percentage bias and p values with Benjamini multiple testing correction. Also shown is the rank order of the CIS in the Kool *et al.* screen.
(TIF)

Table S6 Most frequent intragenic insertions without orientation bias. Unbiased/intragenic CIS locations and target genes identified from the RIM screen, ordered by number of insertions are shown. Also shown are the number of hits in the Retrovirus and Transposon Tagged Cancer Gene Database (RTCGD, Akagi *et al.*, Nucleic Acids Res., 2004, 32: D523–527), and the rank-order position of the gene in terms of peak height from a total of 823 CIS in the Kool *et al.* data set. N/A denotes the absence of the gene in the Kool *et al.* CIS list.
(TIF)

Table S7 CISs displaying loss of selection in Runx2/MYC lymphoma progression compared to end-stage lymphomas. Some of the most strongly selected target genes in the Kool *et al.* meta-analysis of 956 lymphomas are notably under-represented in the Progression CISs. This is illustrated in the table where the most discordant examples are compared by CIS peak height (a measure of number hits and degree of clustering) and rank order. A nil entry (-) means that no CIS was recorded in the progression dataset. For comparison, several prominent targets that are shared are listed below. Grey shading denotes reduced selection compared to the meta-analysis.
(TIF)

Table S8 Top targets unique to Huser *et al.* dataset showing gene name and function, peak height and evidence of that gene's role in human cancer.
(PPTX)

Table S9 List of CISs from GIM set 2, showing chromosomal location and associated genes.
(XLSX)

Table S10 .bed file of all GIM2 alignments, showing chromosomal locations, tumour identity, read counts and strand polarity.
(XLSX)

Table S11 Number of insertions in end-stage lymphomas of three genotypes (wild-type, Runx2, MYC) for the top 25 progression CISs (from analysis 1). The numbers of expanded clones (RIS with >100 reads) are listed in brackets.
(TIF)

Table S12 The number of insertions in end-stage tumours of three genotypes (wild-type, Runx2 and MYC) is shown for the most frequent targets for intragenic insertions in the progression network. Numbers in brackets denote expanded RIS (>100 reads).
(TIF)

Table S13 CIS biased by genotype, sorted by genotype in end-stage lymphomas. Number of RIS are shown alongside number of reads/RIS separated by |. Shading denotes apparent positive selection (reads/RIS>10). In some cases there are clear qualitative differences that are not amenable to statistical comparison due to small numbers of RISs (e.g. Mycn). Significant differences for insertions * at Runx2 between Runx2 and MYC genotypes (P = 0.021) and ** at *Ikzf1* between WT and MYC (P = 0.034) and between Runx2 and MYC (P = 0.04) (Mann-Whitney *U* test).
(TIF)

Table S14 Comparison of Maser *et al.* genomic regions that are amplified or deleted in TKO mouse and human T-ALL with Huser *et al.* CIS regions. Shown are the chromosome locations of the syntenic regions (common MCRs = minimal critical regions) from Maser *et al.*, and the start, end and peak locations as well as peak height of CIS coinciding with these regions. Also shown are gene annotations for CIS and whether the CIS is intragenic or not. The CIS at Gpr132 is both intragenic and upstream of the gene and is labelled +/−. 43/771 CISs map to the syntenic regions which encompass 79.7 Mb of genomic sequences. To assess the probability of this overlap, the mouse genome was considered as a series of 30 kb ‘bins’, the average size of the defined CISs. On this basis, the syntenic regions occupy 2613/89796 bins. The probability of the observed overlap based on the contingency table below is P = <0.0001 (http://vassarstats.net/tab2x2.html).
(TIF)
