# Supplementary figures and images for: Insertional Mutagenesis and Deep Profiling Reveals Gene Hierarchies and a Myc/p53-Dependent Bottleneck in Lymphomagenesis
Source: PLoS Genet. 2014 Feb 27;10(2):e1004167. doi: 10.1371/journal.pgen.1004167 (PMC3937229; doi:10.1371/journal.pgen.1004167)

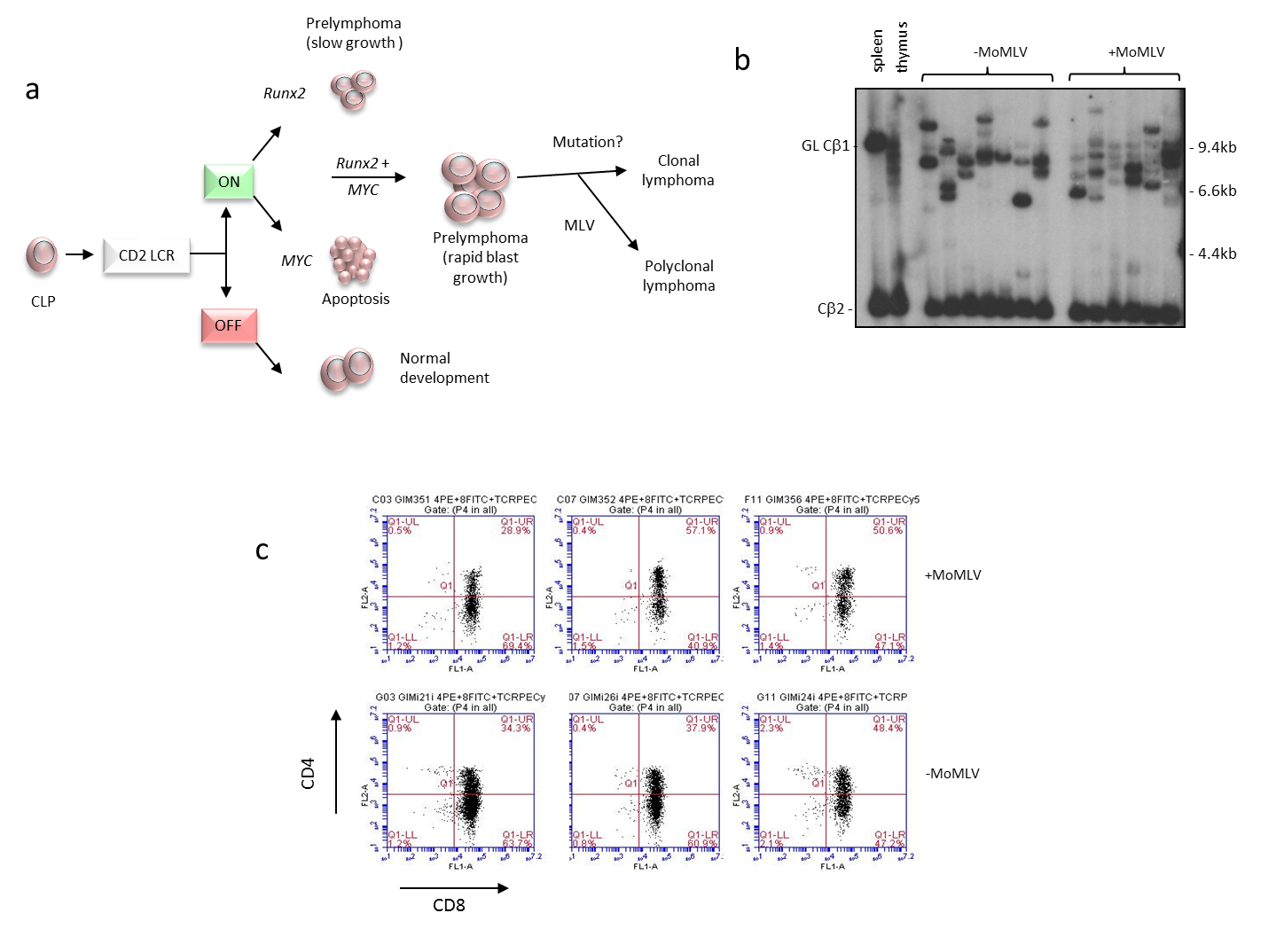

Supplement: Figure S1 — (a) Basic features of the lymphoma model. Expression of either Runx2 or MYC under the control of the CD2 locus control region leads to a low lifetime incidence of T-cell lymphoma. This appears to be due to the variegated activation of the transgenes and counter-selection for expressing cells which either die by apoptosis (MYC) or grow slowly with impaired differentiation (Runx2). The combination of both transgenes cancels these failsafe responses and leads to early onset lymphoma in a 100% of mice [8], [17], [18], [23]. Tumour onset can be accelerated further by neonatal infection with Moloney murine leukaemia virus (MoMLV) [26]. (b) The clonal nature of CD2-Runx2/MYC lymphomas is demonstrated by rearrangements of the T-cell receptor β-chain. Southern blot analysis of 20 mg samples of DNA digested with HindIII and analysed with a Cβ probe. The virtual disappearance of the unrearranged Cβ1 is due to the replacement of non-lymphoid cells by lymphoid cells carrying deletions or rearrangements of Cβ1. As TCR rearrangement can result in productive rearrangement or deletion of Cβ1, dominant clones may be represented by one or two bands. As can be seen, spontaneous tumours in these mice typically display a single major clone, although some evidence of minor clones is present in some cases (-MoMLV). In MoMLV accelerated tumours, there is typically a more complex pattern indicative of greater clonal complexity. Due to the limited sensitivity of Southern blot analyses, clones representing less than 5% of the tumour mass are not detectable. c Phenotypic analysis of CD4 and CD8 expression in primary thymic lymphoma CD2-Runx2/MYC mice. Note that normal thymocytes were almost completely replaced by the characteristic bi-modal tumour cell population (>96–99%). No phenotypic difference was observed in MoMLV-accelerated lymphomas. (TIF) [file pgen.1004167.s001.tif]

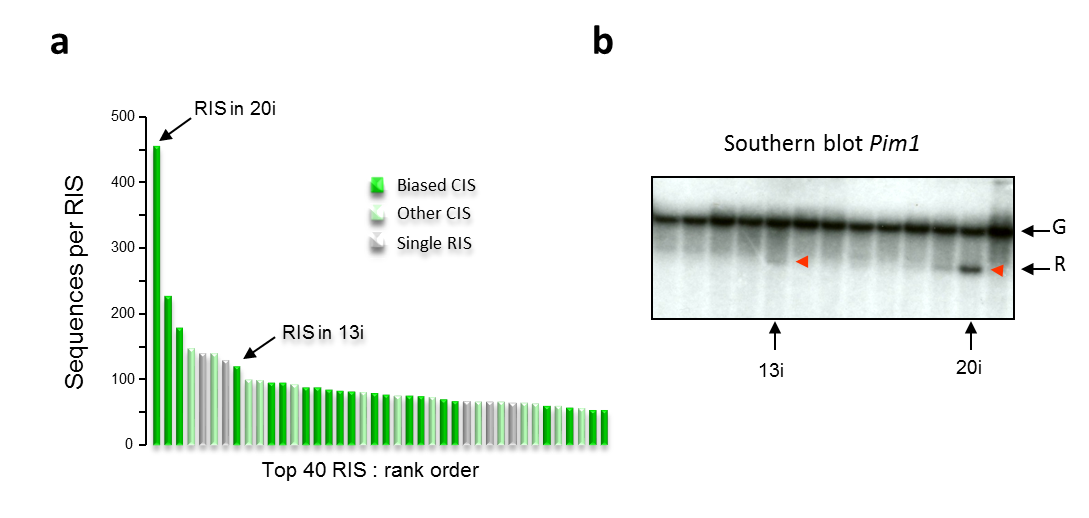

Supplement: Figure S2 — (a) Evidence that expanding clones in virus-accelerated Runx2/MYC lymphomas contain a single provirus. The top 40 RISs (in rank order by number of reads) shows few insertions at isolated RIS far from known target genes(5/40), although these predominate (85%) in the total population of 12,485 RISs. If clonal expansion required two or more hits of proviral insertion, we would expect many more instances of co-amplification of passenger RIS (grey bars). (b) There is a correlation between splinkerette 454 sequence reads and Southern blot detection of rearrangement, with insertions at Pim1 in expanded tumours clones in tumours 20i and 13i being detected by both methods at similar relative efficiency (compare to (a)). G: germ line; R: retrovirus insertion. (TIF) [file pgen.1004167.s002.tif]

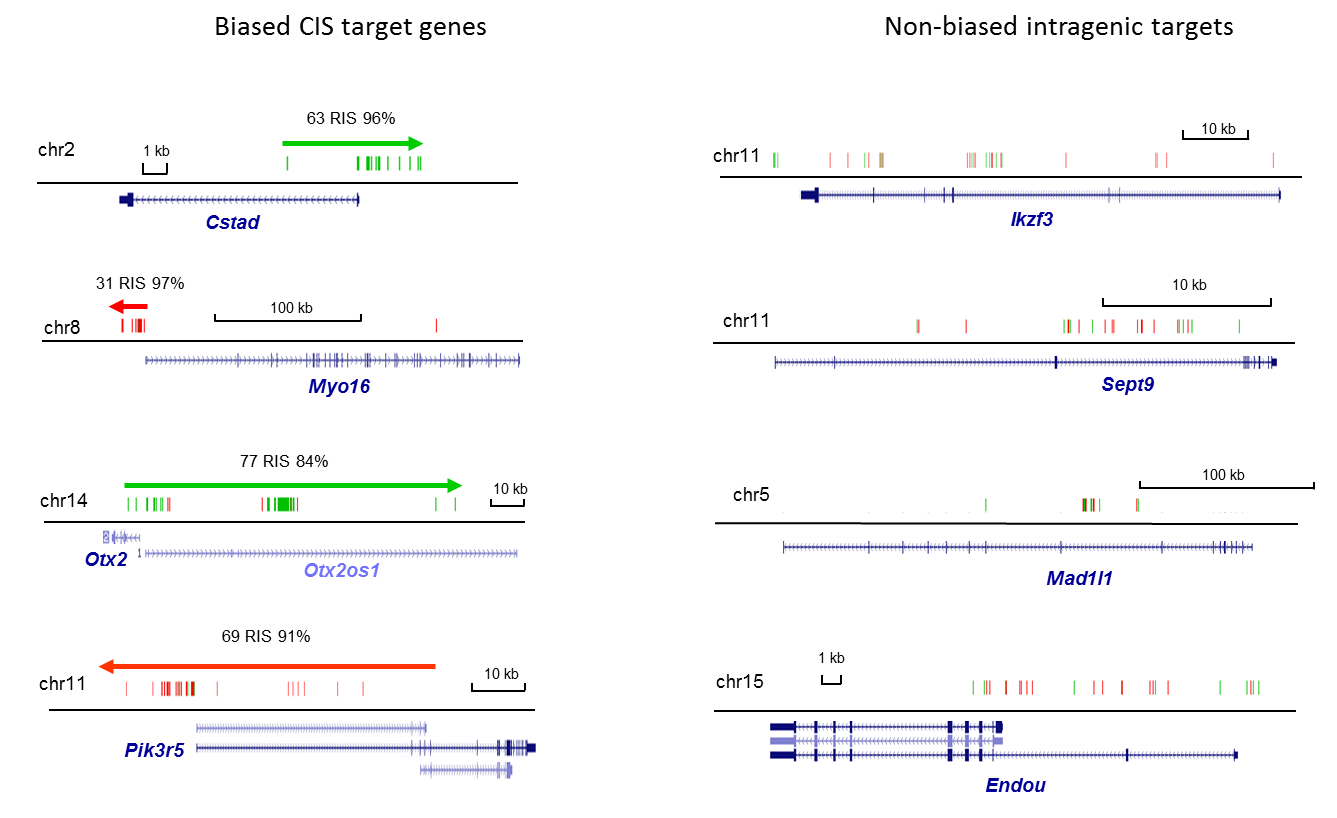

Supplement: Figure S3 — Additional MLV insertion patterns at other biased and non-biased CISs. Each vertical bar represents an individual RIS, red indicates reverse orientation compared to the+strand, green the same orientation. The positions of exons and introns were extracted from the UCSC genome browser (NCBI37/mm9). (TIF) [file pgen.1004167.s003.tif]

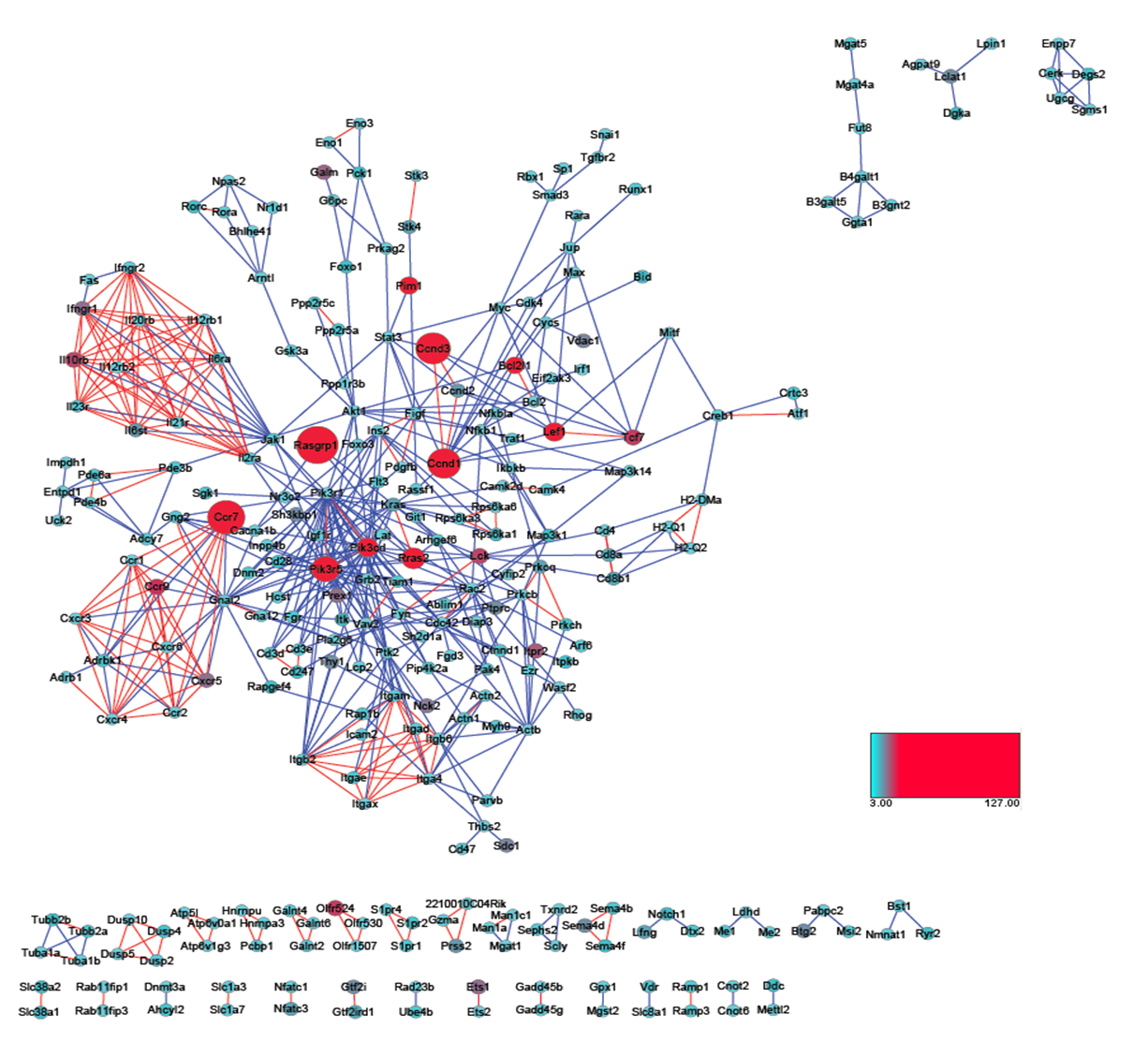

Supplement: Figure S4 — KEGG Cytoscape plot. Genes with RIS counts of 3 or more are visualized in the context of their KEGG pathway interactions using Cytoscape. The KEGG network is based on metanodes. A metanode is a collection of genes that share similar function. Some metanodes only contain a single gene. Links in the KEGG network denote a functional interaction between any of the genes in the two metanodes connected by the link. For visualization purposes the metanodes themselves are not displayed. Consequently, in the resulting graph a link between two genes is present if there is a link between the metanodes in which these genes reside. Blue links are KEGG pathway links, red links connect genes that are in the same metanode in KEGG. Genes that are in the same metanode share functionality (according to KEGG). Note that metanodes are not necessarily consistent across different pathways, which is why some genes that are in the same metanode have a different set of interaction partners. Node colour and size represents the number of RIS attributed to that gene: blue and small circle: 3 RIS, red and large circle: up to a maximum of 127 RIS. (TIF) [file pgen.1004167.s004.tif]

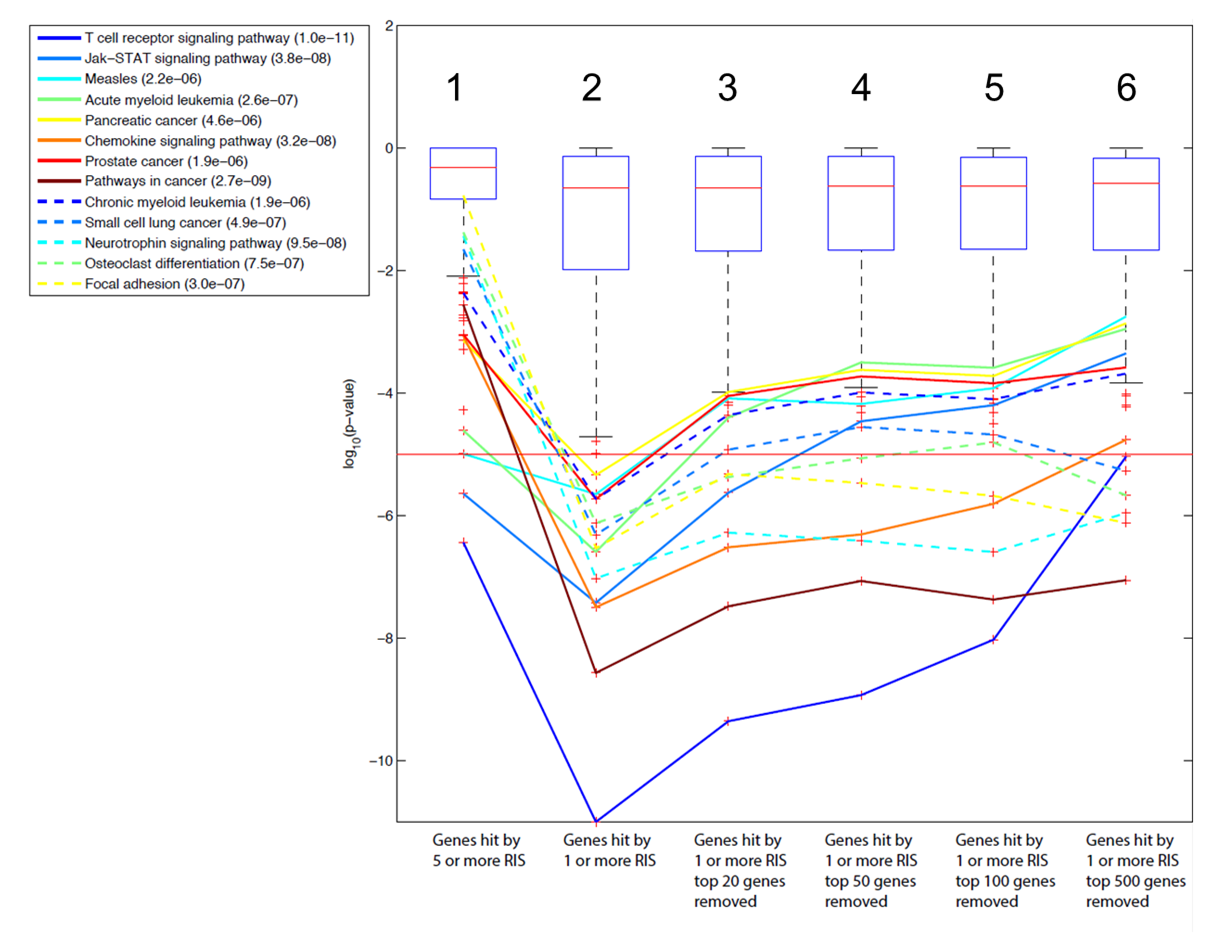

Supplement: Figure S5 — KEGG pathway enrichment analysis. The effects on pathway analysis of limiting gene sets by number of hits or removal of most prominent CISs. This analysis was conducted to test the extent to which oncogenic selection is present throughout the detected RISs. Box plots represent log10 p-values for all pathways in the KEGG database. The legend lists the pathways with a significant p-value (at the 1×10E-5 level) for at least one of the discovery set definitions, with the minimum p-value between parentheses. The leftmost box depicts the log10 p-values of the pathway enrichment when the discovery set is defined as all genes associated with five or more RISs. For the second box from the left the discovery set is defined as all genes with at least one associated RIS. This is also the case for box 3 through 6, but in those discovery sets the top 20, 50, 100 and 500 most frequently targeted genes are removed from the discovery set, respectively. The horizontal red line indicates the 10−5 significance level. Surprisingly, enrichment is more significant when the entire ‘integrome’ is analysed than when restricted to genes that are frequently targeted (by 5+ RIS). Moreover, removal of 20 to 100 ‘top hit’ genes which includes genes common to many of the annotated pathways (e.g. Ccnd, PI3K, Pim gene families) has relatively modest effects on significance scores, while enrichment for pathways in cancer and others survives even the removal of the top 500 genes. These results strongly indicate that either a) the majority of RISs, including those that are not common across multiple tumours, have been subjected to oncogenic selection or b) viral targeting of these pathways is an underlying phenomenon based on integration preference. (TIF) [file pgen.1004167.s005.tif]

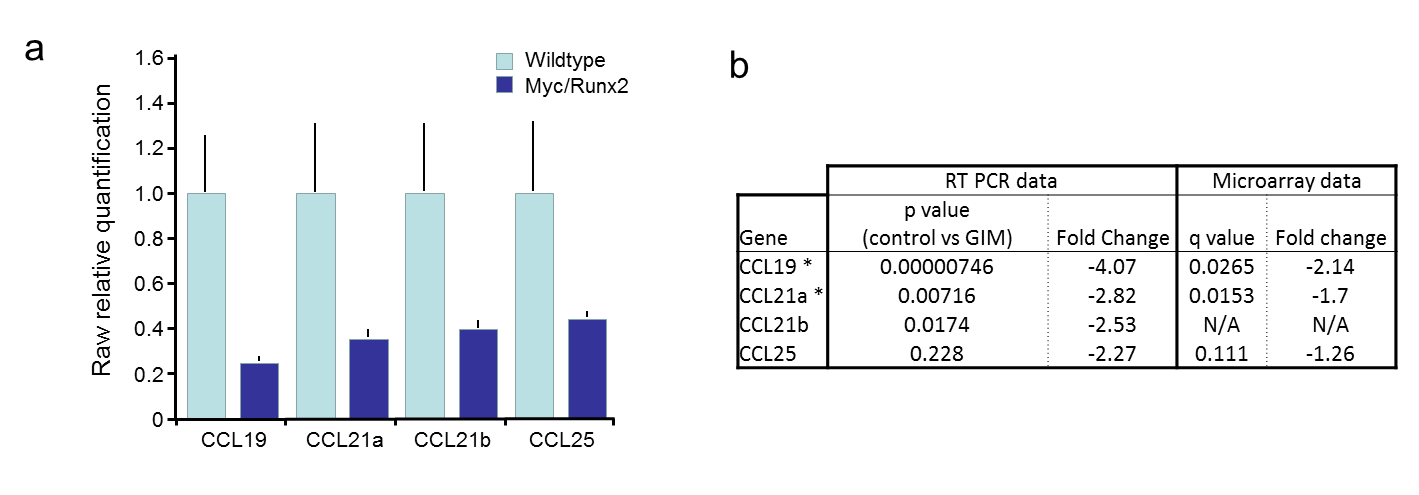

Supplement: Figure S6 — (a) Quantitative real-time PCR validation of key gene changes observed in the microarray. Quantification is relative to house-keeping gene TBP for genes in the CC chemokine family, with fold changes and significance as determined by two-tailed unpaired student's T-test shown in (b) Error bars represent S.E.M. Genes with fold differences reaching a q<0.05 significance threshold in the microarray are noted with an asterisk (*). N/A = gene not present on the microarray. (TIF) [file pgen.1004167.s006.tif]

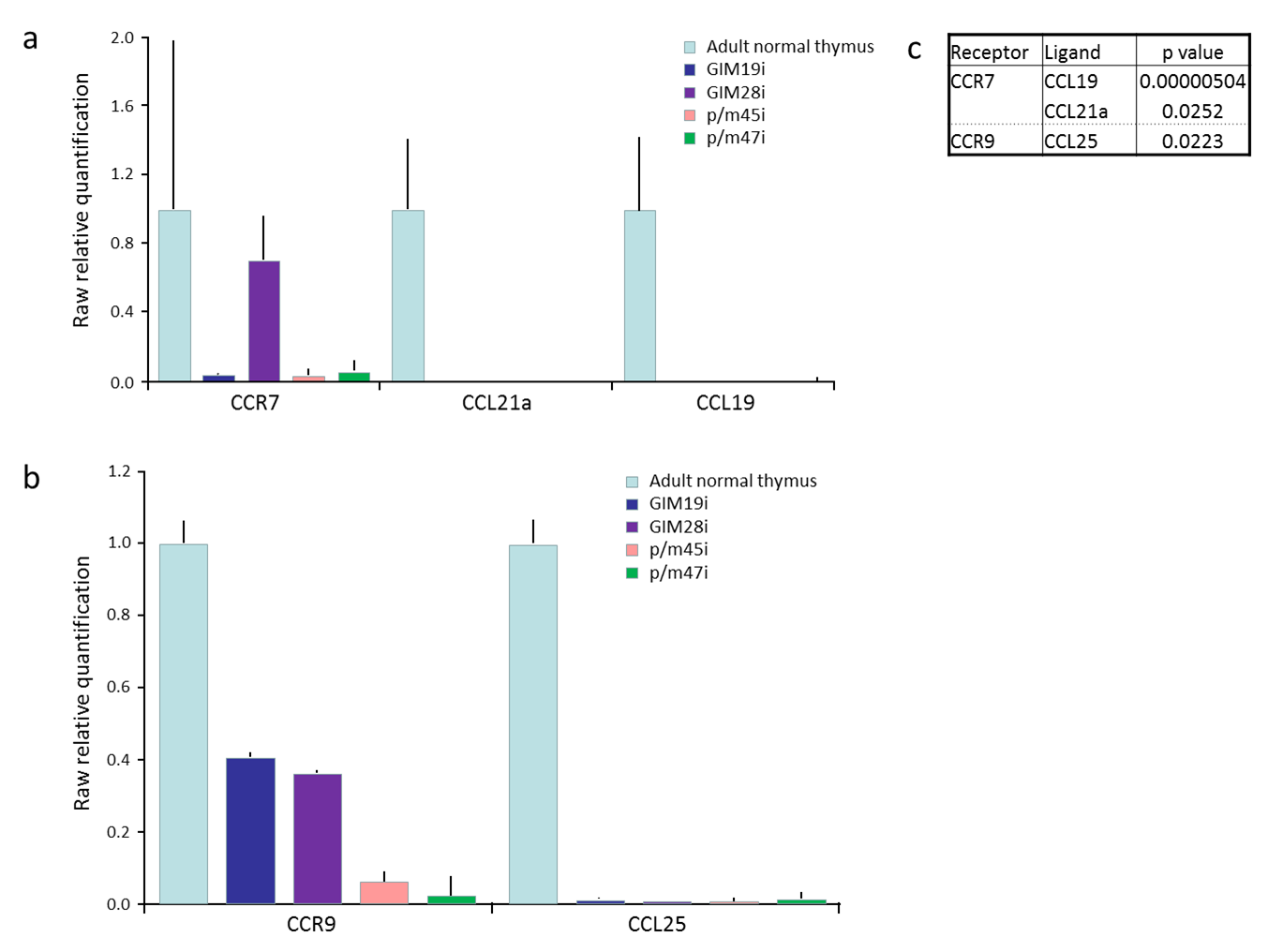

Supplement: Figure S7 — Lack of expression of CC chemokine genes in lymphoma cells from Runx2/MYC and other genetic backgrounds. Quantitative real-time PCR analysis of CC chemokine receptors and ligands for a number of T-cell lymphoma lines from Runx2/MYC (GIM) or p53null/MYC (p/m) backgrounds, expressed relative to adult normal thymus, with HPRT as control. (a) Ccr7 and ligands (b) Ccr9 and ligand. (c) Significance of down-regulation of CC ligands compared to receptors in T-cell lines, determined by two-tailed unpaired student's T test. Errors represent standard error (SEM). (TIF) [file pgen.1004167.s007.tif]

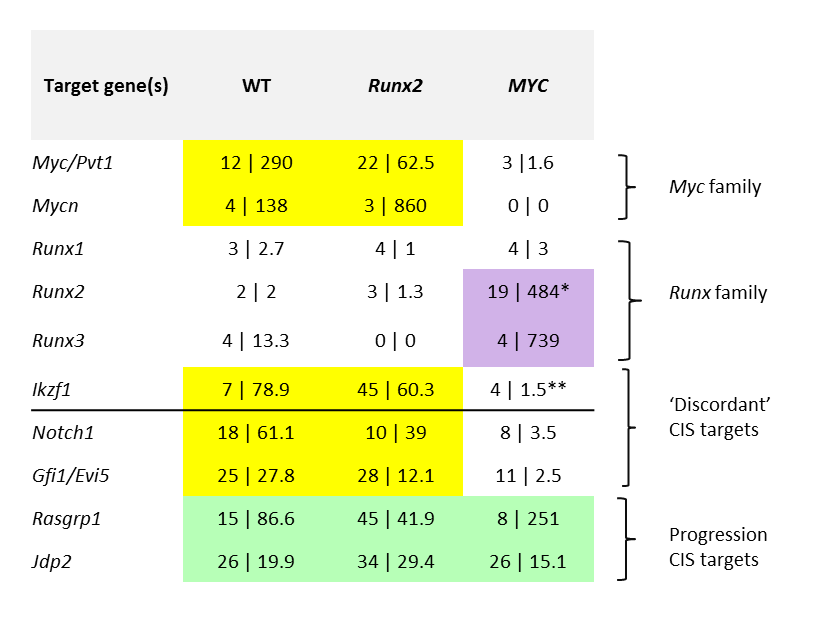

Supplement: Table S13 — CIS biased by genotype, sorted by genotype in end-stage lymphomas. Number of RIS are shown alongside number of reads/RIS separated by |. Shading denotes apparent positive selection (reads/RIS>10). In some cases there are clear qualitative differences that are not amenable to statistical comparison due to small numbers of RISs (e.g. Mycn). Significant differences for insertions * at Runx2 between Runx2 and MYC genotypes (P = 0.021) and ** at Ikzf1 between WT and MYC (P = 0.034) and between Runx2 and MYC (P = 0.04) (Mann-Whitney U test). (TIF) [file pgen.1004167.s020.tif]
